# Augmenting central arterial stiffness following eradication of HCV by direct acting antivirals in advanced fibrosis patients

**DOI:** 10.1038/s41598-018-37829-4

**Published:** 2019-02-05

**Authors:** Pin-Nan Cheng, Ju-Yi Chen, Yen-Cheng Chiu, Hung-Chih Chiu, Liang-Miin Tsai

**Affiliations:** 1 Division of Gastroenterology and Hepatology, Department of Internal Medicine, National Cheng Kung University Hospital, College of Medicine, National Cheng Kung University, Tainan, Taiwan; 2 Division of Cardiology, Department of Internal Medicine, National Cheng Kung University Hospital, College of Medicine, National Cheng Kung University, Tainan, Taiwan

## Abstract

Chronic hepatitis C (CHC) is strongly associated with risks of cardiovascular diseases. The impact of direct acting antiviral (DAA) therapy on central blood pressure remains unclear. This investigation evaluates changes in central blood pressure following DAA therapy. One hundred and two DAA-treated patients were prospectively enrolled. Lipid profiles and pulse wave analysis of brachial artery by cuff sphygmomanometry including augmentation index (AIx), a parameter of central artery stiffness, were evaluated. All of the 102 patients achieved sustained virological response (SVR12). Cholesterol and LDL significantly increased following SVR12. Along with lipid changes, significantly higher central diastolic pressure (78.2 ± 14.2 mm Hg at baseline vs. 83.3 ± 13.9 mm Hg at SVR12, p = 0.011) and AIx (33.0 ± 12.7% at baseline vs. 36.9 ± 12.9% at SVR12, p = 0.012) were only observed in the advanced fibrosis patients. Co-morbid diseases, including hypertension (33.4 ± 13.0% vs. 39.7 ± 12.6%, p = 0.003), abnormal waist circumference (33.8 ± 12.2% vs. 38.0 ± 13.2%, p = 0.027), and metabolic syndrome (34.5 ± 12.1% vs. 39.0 ± 11.2%, p = 0.043) were associated with augmented AIx upon SVR12. The augmented central artery stiffness following viral eradication by DAA therapy may raise the concern of short-term cardiovascular risk in CHC patients.

## Introduction

Hepatitis C virus (HCV) infection is a major health issue globally. Chronic infection leads to a risk of the development liver complications, including liver cirrhosis and hepatocellular carcinoma, and it increases liver-related morbidity and mortality^1^. Additionally, long-standing infection increases the risk of extrahepatic events^2^. With respect to extrahepatic diseases of that are associated with HCV, a strong association exists between HCV infection and cardiovascular diseases, including coronary artery diseases^3^, carotid atherosclerosis^4^, and cerebral vascular diseases^4^.

Pulse wave analysis (PWA) that measures by cuff sphygmomanometry of the brachial artery is used to evaluate changes of waveform morphology throughout the arterial system. The shape of pressure waveform changes from the aorta to distal arteries with constant diastolic pressure but a higher increased systolic pressure in the brachial artery than aorta. This feature is an augmentation of systolic pressure arises from an increase in arterial stiffness as the wave moves away from the aorta^5^. PWA can be used to obtain central blood pressures, augmentation pressure, and the augmentation index (AIx), which are associated with risk of cardiovascular diseases^6,7^. Chronic hepatitis C (CHC) patients are known to exhibit an increased pulse wave velocity^8^. and our earlier investigation demonstrated that a higher arterial stiffness index and a lower compliance index were independently associated with CHC infection^9^. However, little is known about the temporal association of central blood pressures with CHC treatment. Therefore, the relationship between changes in PWA parameters and HCV eradication by direct acting antivirals (DAA) in CHC patients is both interesting and important.

The objectives of this investigation are to evaluate the changes in central blood pressure related parameters with HCV eradication by DAA therapy in CHC patients, and to examine the impact of advanced fibrosis on the parameters in PWA.

## Results

### Baseline characteristics of patients

Table 1 presents the relevant characteristics of the 102 enrolled CHC patients, most of whom were female. The major genotype of HCV was 1b (74.5%). Eight patients had a hepatitis B virus co-infection and all of them had HBV DNA level <2000 IU/mL at baseline. Advanced fibrosis was detected in 76 patients, who tended to have a significantly higher age, lower albumin level, lower estimated glomerular filtration rate (eGFR), and lower platelet count (all p < 0.05). Twenty-five patients had a history of hepatocellular carcinoma and all of those had advanced fibrosis. The incidences of diabetes mellitus, hypertension, chronic kidney disease (defined as estimated glomerular filtration rate, eGFR <60 ml/min/1.73 m^2^), dyslipidemia, and coronary artery disease did not differ between patients with non-advanced fibrosis and those with advanced fibrosis.

**Table 1:** Baseline characteristic of the 102 patients.

Variable	ALL N = 102	Non-advanced fibrosis N = 26	Advanced fibrosis N = 76	p value*
Age (years)	66.0 ± 10.7	60.3 ± 12.6	68.0 ± 9.3	0.007
Male Gender	34 (33.3%)	9	25	0.872
Body mass index (kg/m^2^)	25.3 ± 3.7	25.9 ± 4.6	25.1 ± 3.4	0.396
Waist circumference (cm)	85.3 ± 10.3	83.4 ± 11.1	86.0 ± 1.0	0.264
Hip circumference (cm)	97.4 ± 8.3	98.9 ± 9.0	96.9 ± 8.0	0.303
HCV RNA (log_10_ IU/mL)	6.1 ± 0.7	6.0 ± 0.7	6.2 ± 0.7	0.110
HCV Genotype				
1a	8 (7.8%)	3	5	0.085
1b	76 (74.5%)	13	63	
1	1 (1.0%)	0	1	
2	15 (14.7%)	9	6	
4	1 (1.0%)	0	1	
6	1 (1.0%)	1	0	
Albumin (g/L)	4.2 ± 0.3	4.4 ± 0.2	4.2 ± 0.3	<0.001
AST (U/L)	72.6 ± 43.7	57.7 ± 42.2	77.7 ± 43.3	0.043
ALT (U/L)	96.0 ± 89.0	100.2 ± 124.7	94.5 ± 74.0	0.780
Total Bilirubin (mg/dL)	0.8 ± 0.3	0.7 ± 0.3	0.8 ± 0.3	0.280
Creatinine (mg/dL)	0.9 ± 0.9	0.66 ± 0.19	0.93 ± 1.01	0.187
eGFR (mL/min/1.73 m^2^)	80.7 ± 16.9	86.8 ± 10.8	78.7 ± 18.2	0.007
Hemoglobin (g/dL)	13.7 ± 1.6	14.0 ± 1.1	13.6 ± 1.7	0.268
Platelet count (x10^9^/L)	153.3 ± 60.8	204.8 ± 42.6	135.7 ± 56.0	<0.001
Prothrombin time, INR	1.05 ± 0.08	1.01 ± 0.06	1.06 ± 0.08	0.002
Cholesterol (mg/dL)	165.2 ± 28.9	173.5 ± 30.2	162.2 ± 28.1	0.088
Triglyceride (mg/dL)	102.3 ± 38.2	92.0 ± 33.9	105.9	0.112
LDL	101.6 ± 26.2	105.8 ± 25.2	100.2 ± 26.5	0.344
HDL	56.4 ± 18.2	60.8 ± 15.7	54.8 ± 18.9	0.150
Insulin (mU/L)	14.3 ± 6.5	12.6 ± 5.2	14.9 ± 6.8	0.122
HbA1c (%)	5.8 ± 0.9	5.6 ± 0.8	5.9 ± 1.0	0.289
HOMA-IR	0.20 ± 0.09	0.17 ± 0.07	0.21 ± 0.09	0.059
HOMA-β (%)	145.9 ± 91.8	139.9 ± 78.2	148.0 ± 96.0	0.703
Hepatitis B virus infection	8 (7.8%)	5	3	0.024
Hepatocellular carcinoma history	25 (24.5%)	0	25	<0.001
Diabetes mellitus	30 (29.4%)	4	26	0.084
Hypertension	45(44.1%)	10	35	0.501
Dyslipidemia	21 (19.8%)	4	17	0.579
Coronary artery disease	8 (7.8%)	1	7	0.676
Chronic kidney disease	13 (12.7%)	3	10	1.000
Smoking	7 (6.9%)	0	7	0.186
DAA regimen				
PrOD ± Ribavirin	69	9	60	
Sofosbuvir/Daclatasvir ± Ribavirin	14	8	6	
Sofosbuvir/Ribavirin	1	1	0	
Ledipasvir/Sofosbuvir	5	5	0	
Grazoprevir/Elbasvir	13	3	10	

*Comparison was performed between non-advanced fibrosis and advanced fibrosis patients.

AST: aspartate aminotransferase; ALT: alanine aminotransferase; eGFR: estimated glomerular filtration rate; LDL, low-density lipoprotein of cholesterol; HDL, high-density lipoprotein of cholesterol; ProD, paritaprevir/ritonavir-ombitasvir and dasabuvir.

### Response to treatment and changes in lipid/sugar profiles

Nighty patients (88.2%) had undetectable serum HCV RNA at week 4 of DAA therapy. All 102 patients (100%) achieved SVR12. Liver stiffness declined significantly from 15.4 ± 11.2 kPa at baseline to 11.8 ± 8.6 kPa at SVR12 (p < 0.001). At baseline, levels of insulin, HbA1c, HOMA-IR, HOMA-β, cholesterol, triglyceride, LDL, and HDL were similar across fibrosis stage (Table 1). After SVR12 achieved, cholesterol, triglyceride, LDL, and HOMA-β levels were significantly higher than baseline levels (Table 2). Significant increases in cholesterol and LDL levels were observed in both non-advanced fibrosis and advanced fibrosis patients.

**Table 2:** Comparison of lipid, arterial pressures, and liver/arterial stiffness associated parameters of non-advanced fibrosis and advanced fibrosis patients.

	All, n = 102			Non-advanced fibrosis, n = 26			Advanced fibrosis, n = 76		
	Baseline	SVR12	p	Baseline	SVR12	p	Baseline	SVR12	p
Liver stiffness (kPa)	15.4 ± 11.2	11.8 ± 8.6	<0.001	6.1 ± 1.5	5.5 ± 1.5	0.080	15.8 ± 10.7	13.9 ± 9.1	0.004
Cholesterol (mg/dL)	165.2 ± 28.9	182.8 ± 33.4	<0.001	173.5 ± 30.2	192.5 ± 32.1	0.002	162.6 ± 28.1	179.4 ± 33.4	<0.001
Triglyceride (mg/dL)	102.7 ± 38.2	112.6 ± 49.2	0.013	93.3 ± 34.0	113.5 ± 53.6	0.020	105.9 ± 39.2	112.3 ± 48.0	0.153
LDL (mg/dL)	101.6 ± 26.2	117.8 ± 31.5	<0.001	105.8 ± 25.2	123.7 ± 28.5	0.002	100.2 ± 26.5	115.7 ± 32.5	<0.001
HDL (mg/dL)	56.4 ± 18.2	56.7 ± 15.4	0.855	60.8 ± 15.7	60.5 ± 15.6	0.866	54.8 ± 18.9	55.3 ± 15.3	0.781
Insulin (mU/L)	14.3 ± 6.5	13.4 ± 6.6	0.192	12.6 ± 5.2	11.8 ± 5.8	0.535	14.9 ± 6.8	14.0 ± 65.8	0.256
HbA1c (%)	5.8 ± 0.9	5.7 ± 0.8	0.365	5.6 ± 0.8	5.6 ± 0.6	0.879	5.9 ± 1.0	5.8 ± 0.8	0.371
HOMA-IR	0.20 ± 0.09	0.19 ± 0.10	0.217	0.17 ± 0.07	0.17 ± 0.09	0.733	0.21 ± 0.09	0.20 ± 0.10	0.225
HOMA-β (%)	145.9 ± 91.8	130.5 ± 86.3	0.035	139.9 ± 78.2	125.8 ± 72.8	0.357	148.1 ± 96.7	132.2 ± 91.1	0.057
hsCRP (mg/dL)	1.17 ± 2.57	1.52 ± 4.12	0.436	0.57 ± 0.58	1.19 ± 3.02	0.259	1.38 ± 2.94	1.64± 4.45	0.657
Systolic pressure (mm Hg)	139.8 ± 21.0	140.3 ± 21.1	0.813	138.4 ± 25.6	131.2 ± 208	0.027	14.02 ± 19.8	143.4 ± 20.4	0.223
Diastolic pressure (mm Hg)	78.4 ± 11.1	80.1 ± 12.1	0.158	79.5 ± 9.6	76.5 ± 10.1	0.096	78.1 ± 11.6	81.3 ± 12.6	0.024
Mean arterial pressure (mm Hg)	98.6 ± 14.0	99.1 ± 15.2	0.755	99.3 ± 14.9	94.7 ± 12.7	0.039	98.4 ± 13.8	100.6 ± 15.7	0.252
Heart rate (beat/min)	70.5 ± 11.1	70.5 ± 11.4	0.976	71.8 ± 11.1	71.6 ± 14.2	0.936	70.1 ± 11.1	70.1 ± 10.3	0.993
Central systolic pressure (mm Hg)	127.5 ± 18.9	128.8 ± 19.1	0.514	126.7 ± 22.4	120.5 ± 18.2	0.039	127.8 ± 17.8	131.6 ± 18.6	0.109
Central diastolic pressure (mm Hg)	78.9 ± 13.2	81.8 ± 13.3	0.062	80.9 ± 9.9	76.5 ± 10.1	0.047	78.2 ± 14.2	83.3 ± 13.9	0.011
Central pulse pressure (mm Hg)	47.9 ± 13.5	47.4 ± 13.2	0.702	45.8 ± 14.8	43.0 ± 13.7	0.181	48.6 ± 13.1	48.9 ± 12.8	0.810
Augmentation pressure (mm Hg)	16.5 ± 9.2	17.9 ± 9.2	0.114	15.7 ± 9.7	15.5 ± 7.9	0.893	16.8 ± 9.0	18.7 ± 9.5	0.073
Augmentation index (%)	32.8 ± 12.6	36.4 ± 12.4	0.005	32.2 ± 12.6	34.8 ± 11.0	0.206	33.0 ± 12.7	36.9 ± 12.9	0.012

### Brachial and central blood pressures

**Figure 1:**
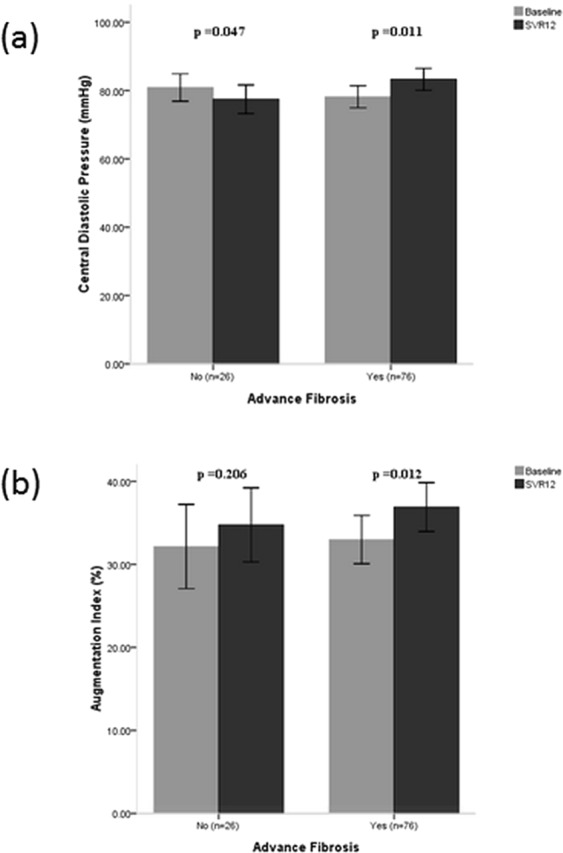
Comparisons of central diastolic pressure and augmentation index before. DAA therapy and following SVR12. Bars represent mean levels and lines inside the box plot 95% of confidence interval. (**a**) Changes of central diastolic pressure in patients with non-advanced fibrosis vs. advanced fibrosis; (**b**) Changes of augmentation index in patients with non-advanced fibrosis vs. advanced fibrosis.

**Figure 2:**
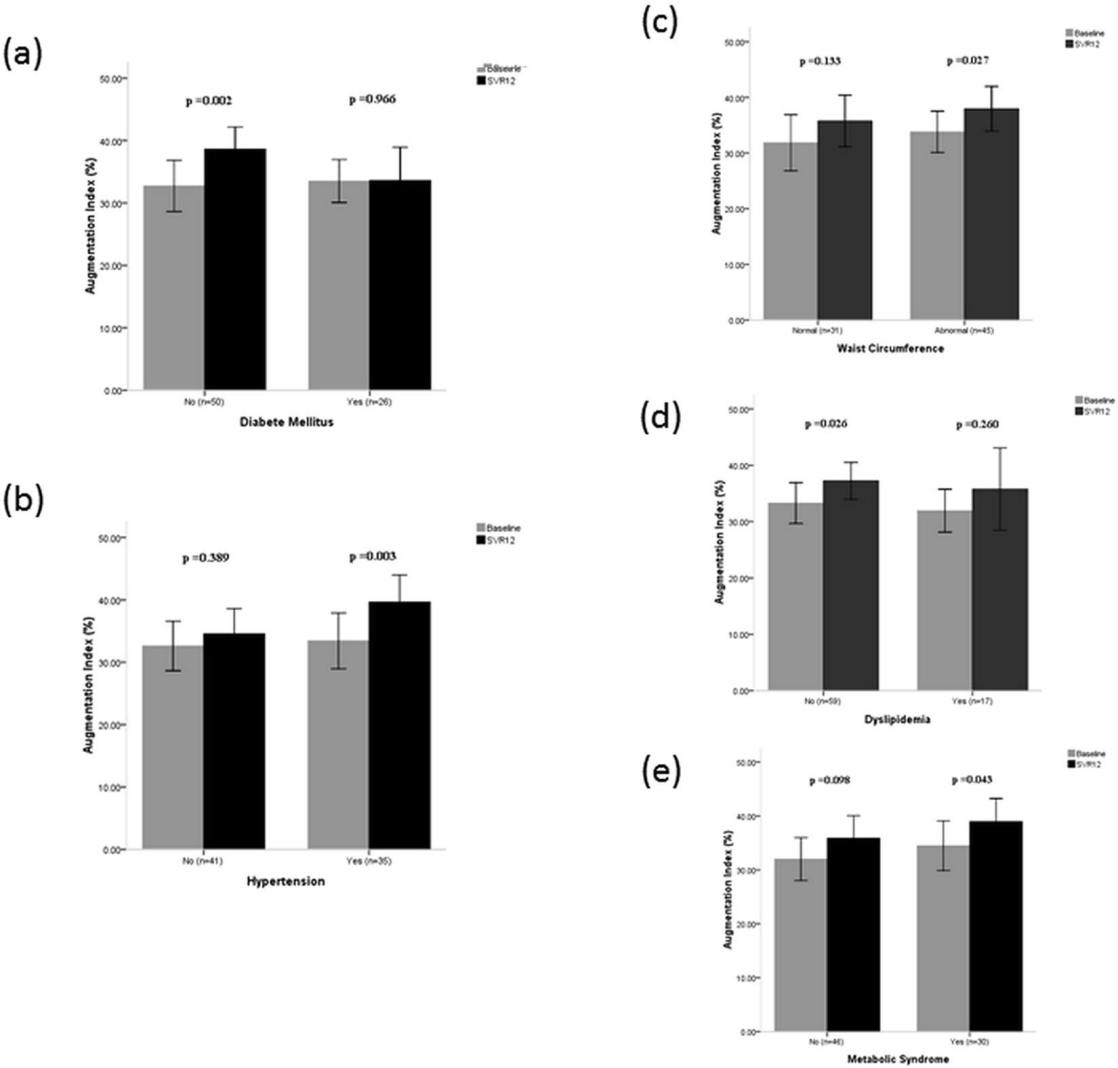
Comparisons of augmentation index following SVR12 in CHC patients with advanced fibrosis and pre-existed co-morbid diseases. Bars represent mean levels and lines inside the box plot 95% of confidence interval. (**a**) diabetes mellitus; (**b**) hypertension; (**c**) waist circumference; (**d**) dyslipidemia; (**e**) metabolic syndrome.

Among the 102 patients, baseline hsCRP, systolic pressure, diastolic pressure, mean arterial pressure, heart rate, central systolic pressure, central diastolic pressure, augmentation pressure, and AIx did not differ between patients with non-advanced fibrosis and those with advanced fibrosis (All p > 0.05). AIx was correlated with age (r = 0.232, p = 0.019) and was significantly higher in females than in males (34.6 ± 12.7% vs. 29.0 ± 11.8%, p = 0.033). PWA related parameters before and after HCV eradication were compared. Of note, all of these parameters tended to be higher at SVR12 than before DAA therapy. Only AIx were significantly increased (Table 2). Comparison of the PWA related parameters between baseline and SVR12 was investigated, and it was found thatcentral diastolic pressure significantly increased both in advanced and non-advanced fibrosis patients and AIx significantly increased only in advanced fibrosis patients (Fig. 1). Brachial diastolic pressure changed similarly. In contrast, brachial systolic pressure, mean arterial pressure, and central systolic pressure decreased significantly in non-advanced fibrosis patients. Accordingly, the association of co-morbid diseases with AIx in advanced fibrosis patients was analyzed (Fig. 2). After SVR12 was achieved, AIx in patients with hypertension (33.4 ± 13.0% at baseline vs. 39.7 ± 12.6% at SVR12, p = 0.003), abnormal waist circumference (33.8 ± 12.2% at baseline vs. 38.0 ± 13.2% at SVR12, p = 0.027), and metabolic syndrome (34.5 ± 12.1% at baseline vs. 39.0 ± 11.2% at SVR12, p = 0.043) was significantly higher. For patients with diabetes mellitus (DM), AIx was unchanged by HCV eradication (33.5 ± 8.5% at baseline vs. 33.6 ± 13.1% at SVR12, p = 0.966). In contrast, non-DM patients exhibited increased AIx (32.7 ± 14.5% at baseline vs. 38.6 ± 12.5% at SVR12, p = 0.002).

Similar to patients with DM, significantly higher AIx was present in patients without non-dyslipidemia (33.3 ± 13.9% at baseline vs. 37.2 ± 12.6% at SVR12, p = 0.026) rather than in those with dyslipidemia (31.9 ± 7.4% at baseline vs. 35.8 ± 14.2% at SVR12, p = 0.260). Other associated factors, including tobacco use, pre-existing chronic kidney disease, and pre-existing coronary artery diseases, were also analyzed. No significant changes of AIx were observed in patients with tobacco use (n = 7, 33.7 ± 9.7% at baseline vs. 33.1 ± 15.4% at SVR12, p = 0.932), pre-existing chronic kidney disease (n = 10, 29.7 ± 15.9% at baseline vs. 37.4 ± 14.5% at SVR12, p = 0.246), and pre-existing coronary artery disease (n = 7, 31.6 ± 15.8% at baseline vs. 30.7 ± 14.3% at SVR12, p = 0.909) following viral clearance. Multivariate linear regression analysis was performed to identify metabolic factors associated with increased AIx. The only independent metabolic factor was absence of DM (p = 0.05). Other factors including dyslipidemia (p = 0.657), hypertension (p = 0.184), and abnormal waist circumference (p = 0.823) did not show significance.

## Discussion

DAA is a short-duration and highly efficient treatment for chronic hepatitis C; viral eradication is accompanied by lipid changes. In this investigation, the cholesterol and LDL levels of patients with non-advanced fibrosis and those with advanced fibrosis were significantly increased upon SVR12. These results are consistent with those in previous reports^10,11^. Our earlier study revealed that DAA treatment increased the loading capacity of triglyceride and cholesterol per very low-density lipoprotein of cholesterol particle, which transports lipid from liver to circulating blood^12^. The cholesterol loading capacity of LDL in the plasma also increased. The rapid changes in cholesterol and LDL levels were probably caused by increased delivery of lipid from liver to circulating blood following viral eradication by DAA therapy.

LDL is strongly associated with a risk of cardiovascular diseases^13^. One recent study investigated the association of LDL with blood pressure and found that a high LDL level in non-statin users was associated with increased brachial and central blood pressures, with the greatest effect on central diastolic pressure^14^. LDL is known to affect detrimentally endothelial function, and elevated serum LDL levels result in higher vascular tone via upregulation of vascular Angiotensin II receptor gene expression by LDL in vascular smooth muscle cells^15^ and a reduction in the intracellular concentration of nitric oxide in endothelial cells^16^. These pathways may be consistent with findings of this investigation that increased LDL levels are associated mostly with an elevated steady component of central blood pressure, which is dominated by arterial resistance^17,18^. In this investigation, PWA of the brachial artery was used to obtain central (aortic) blood pressure and AIx. AIx is derived as augmentation pressure divided by central pulse pressure. An important finding of this investigation is the significant increase of AIx, a parameter of aortic stiffness, upon DAA therapy and the achievement of SVR12. Therefore, we speculate that the increased AIx in CHC patients who cleared HCV as a result of a short period of DAA therapy is partially, at least, mediated by increased LDL levels, which increased central diastolic pressure and reduced central pulse pressure. A mechanism that is not LDL-related may be involved. HCV-induced inflammation may have a role. In this investigation, the unchanged hsCRP upon SVR12 revealed a persistent inflammation reaction following HCV eradication. A cytokine and chemokine study showed that successful DAA therapy reduced the levels of up-regulated inflammation mediators, but not recovered to those of healthy controls^19^. Some mediators did not recover, or even remained elevated during or after therapy^20^. The complex presentations may predispose patients to persistent inflammation, even following HCV eradication. Along with increased LDL, persistent inflammation following HCV eradication may have a supplemental role in increased central diastolic pressure and aortic stiffness.

A significant increase in aortic stiffness was observed only in patients with advanced fibrosis, and not in those with non-advanced fibrosis. In this investigation, patients with advanced liver fibrosis were older than those with non-advanced fibrosis. However, before DAA therapy, their AIx values did not differ (32.2 ± 12.6% vs. 33.0 ± 12.7%, p = 0.773). Cholesterol and LDL levels increased significantly, regardless of fibrosis stage. However, brachial and central diastolic pressures were significantly elevated only in advanced fibrosis patients, indicating that the detrimental effects of LDL on central blood pressure and aortic stiffness seemed to be greater in advanced fibrosis patients. Co-existing diseases or metabolic syndrome were also associated with higher AIx in advanced fibrosis patients. In advanced fibrosis patients, hypertension, abnormal waist circumference, and metabolic syndrome all increased aortic stiffness following viral eradication. However, AIx in patients with DM did not differ before therapy and upon SVR12. A lesser degree of impairment of left ventricular systolic function^21^, blunted response of central blood pressure to glucose^22^, and lower reflection wave magnitude as a result of a reduction of the impedance mismatch of the large artery and distal arteries^23^ may have contributed to the unchanged AIx in patients with DM following viral eradication. All of the DM patients were treated by oral hypoglycemic agents or insulin therapy. Controlling of sugar was not likely to be beneficial to aortic stiffness following SVR12 in this investigation. Similar to patients with DM, significantly higher AIx was observed only in patients with non-dyslipidemia patients. In this investigation, all patients with dyslipidemia took statin. For statin users, reduction of LDL level was associated with a fraction of the reduction of blood pressure and seemed to be associated mostly with the improvement in central steady (diastolic) pressure^14^, and then contributed to a lesser degree of changes in AIx following eradication of HCV. In this investigation, patients with hypertension all treated by anti-hypertensive drugs and also showed higher aortic stiffness than non-hypertension patients that indicated the possible detrimental impacts on diseased arteries of hypertensive patients. Other factors including tobacco use, pre-existing chronic kidney disease, and coronary artery disease did not show impact on AIx following viral clearance. However, the patient number was small that could not make solid conclusion in this investigation.

The long-term implications of the above findings remain to be elucidated. An increase in AIx, a parameter of aortic stiffness, theoretically causes an increase in the afterload of heart and boosts aortic and left ventricular pressure in systole by causing the early return of the reflection wave^24^. These effects may degrade cardiac function. Furthermore, an increase in aortic stiffness reduces aortic pressure during diastole reducing the coronary perfusion pressure and thereby predisposing patients to myocardial ischemia^25^. Patients with pre-existing heart failure or coronary artery disease may be at a risk of developing associated events during treatment and a short follow-up period. Experiences of serious adverse events during or after DAA therapy, especially cardiovascular events, which had previously not been recognized as being related to DAA therapy should be re-examined. Patients with simultaneous advanced fibrosis and disease background of cardiovascular or metabolic diseases that may predispose them to deterioration of cardiovascular function should be closely monitored throughout DAA treatment and a short post-therapy follow-up period.

This study has limitations. The follow-up period was short. Lipid and arterial stiffness should be measured over a longer period after SVR12 is achieved. Based on the beneficial effects of interferon/ribavirin treatment on cardiovascular risk^26^, long-term findings concerning central arterial stiffness may differ from the short-term observations in this study and warrant further investigation. In this investigation, associated endothelial or cytokine factors following the course of DAA therapy were not evaluated. Further studies concerning these issues are needed. Finally, the number of patients with non-advanced fibrosis is limited, more patients enrolment is required to obtain more solid results.

In summary, the data herein suggest that LDL levels increase, along with aortic stiffness, shortly after the eradication of HCV by DAA treatment. These results seem to contradict current knowledge of the long-term beneficial effects of curing HCV on cardiovascular risks. Further large-scale and long-term prospective investigations are required to address this interesting and important issue.

## Methods

### Patients

Patients who had CHC and underwent DAA therapy at the National Cheng Kung University Hospital from February 2017 to October 2017 were prospectively enrolled in this study. CHC was defined as positivity for anti-HCV antibody for more than six months with the presence of serum HCV RNA. Medical history, including co-morbid diseases, alcohol consumption, smoking status, co-medications, and CHC treatment history were reviewed. Eligible patients were followed up for 12 weeks following the completion of DAA therapy. Fasting blood sampling was carried out, and measurements of liver stiffness, waist circumference, body weight and body height made before therapy and at SVR12. Waist circumference ≥90 cm in males or ≥80 cm in females was regarded as abnormal. Patients who exhibited three of the five following characteristics were diagnosed as having metabolic syndrome; abnormal waist circumference, systolic blood pressure ≥130 mm Hg or diastolic blood pressure ≥85 mm Hg or currently taking blood pressure-lowering agents, high-density lipoprotein of cholesterol <40 mg/dL in males or <50 mg/dL in females, fasting blood sugar ≥100 mg/dL or currently taking diabetes medications, and triglyceride ≥150 mg/dL. In addition, Homeostasis Model Assessment (HOMA) of insulin resistance (IR) and beta cell function (β) were evaluated. The equation of HOMA-IR and HOMA-β was (fasting sugar × insulin)/405 and (360 × insulin)/(fasting sugar-63), respectively. Advanced liver fibrosis was defined as liver stiffness ≥9.5 kPa, measured by transient elastography (FibroScan®, Echosens, France) or Fibrosis-4 score ≥3.25. The Research Ethics Committees of the National Cheng Kung University Hospital approved this investigation (No: A-ER-105–479), which was conducted according to the guidelines of the International Conference on Harmonization for Good Clinical Practice. All patients provided written informed consent before enrollment.

### Laboratory examinations

Blood samples were stored at −70 °C until testing. High-sensitivity C-reactive protein (hsCRP), lipid profiles including cholesterol, triglyceride, low-density lipoprotein of cholesterol (LDL), and high-density lipoprotein of cholesterol (HDL), and sugar profiles including fasting sugar, glycemic hemoglobin (HbA1c), and insulin were obtained both before therapy and 12 weeks after the end of treatment. Quantitation of HCV RNA was using the real-time polymerase chain reaction (Abbott RealTime HCV quantitative assay; Abbott Molecular Inc., Des Plaines, IL, USA). Sustained virological response (SVR12) was defined as undetectable HCV RNA at 12 weeks after the end of DAA therapy.

### Acquisition of peripheral pressure waveform signals, pressure wave reflection, and augmentation index

All tests were carried out in the morning (9:00~11:00am). Before any tests were performed, all subjects rested in a sitting position for five minutes in a quiet, temperature-controlled (26° ± 1 °C) room. PWA was carried out using a SphygmoCor XCEL PWA (AtCor, Sydney, Australia). A conventional brachial cuff on the right upper arm of each subject was used to measure brachial systolic and diastolic pressures, and a brachial waveform was thus captured. The brachial waveform was then analyzed using SphygmoCor to provide a central aortic waveform. Central blood pressure measurements, such as those of central aortic systolic blood pressure, central pulse pressure and AIx, were made. AIx measures the degree to which the peak of a measured pressure wave exceeds the peak of the incident pressure wave owing to the addition of the reflected pressure wave. The AIx depends on the timing and magnitude of the reflected waveform and is affected by the compliance and structure of vessels distal to the site of measurement. The SphygmoCor system uses the more common AIx as the augmentation pressure (central systolic pressure minus inflection pressure) divided by the pulse pressure (central systolic minus central diastolic pressure), expressed as a percentage. The AIx ratio was controlled at a hear rate of 75 beats/min.

### Statistical analyses

The data were expressed as mean plus standard deviation. Continuous variables were compared by the Student t test and categorical variables were compared using the Chi-Square test or Fisher’s exact test. Comparisons of lipid profiles, sugar profiles, hsCRP, and parameters of arterial stiffness before treatment and 12 weeks following the end of treatment were carried out using the paired t test. The correlation between AIx and age was evaluated. Analysis of the associated metabolic factors of increased AIx was carried out by multivariate linear regression analysis. All tests were two-tailed. A p value of less than 0.05 was considered to indicate statistically significance.
